# Characterization of the distributions of collagen and PGs content in the decellularized book-shaped enthesis scaffolds by SR-FTIR

**DOI:** 10.1186/s12891-021-04106-x

**Published:** 2021-03-01

**Authors:** Qiang Shi, Can Chen, Muzhi Li, Yang Chen, Yan Xu, Jianzhong Hu, Jun Liu, Hongbin Lu

**Affiliations:** 1 Department of Sports Medicine, Xiangya Hospital, Central South University, Changsha, 410008 Hunan China; 2 Key Laboratory of Organ Injury, Aging and Regenerative Medicine of Hunan Province, Changsha, 410008 Hunan China; 3 Xiangya Hospital-International Chinese Musculoskeletal Research Society Sports Medicine Research Centre, Changsha, 410008 Hunan China; 4 Department of Orthopedics, Xiangya Hospital, Central South University, Changsha, 410008 Hunan China; 5 Department of Spine Surgery, Xiangya Hospital, Central South University, Changsha, 410008 Hunan China; 6 Department of limbs (foot and hand) microsurgery, Affiliated Chenzhou No.1 People’s Hospital, Southern Medical University, Chenzhou, 423000 Hunan China

**Keywords:** SR-FTIR, Decellularized book-shaped enthesis scaffolds, Bone-tendon interface, Rabbit rotator cuff

## Abstract

**Background:**

Bone-tendon interface (enthesis) plays a pivotal role in relaxing load transfer between otherwise structurally and functionally distinct tissue types. Currently, decellularized extracellular matrix (DEM) from enthesis provide a natural three-dimensional scaffold with tissue-specific orientations of extracellular matrix molecules for enthesis regeneration, however, the distributions of collagen and PGs content in the decellularized book-shaped enthesis scaffolds from rabbit rotator cuff by SR-FTIR have not been reported.

**Methods:**

Native enthesis tissues (NET) harvested from rabbit rotator cuff were sectioned into cuboid (about 30 mm × 1.2 mm × 10 mm) for decalcification. The decellularized book-shaped enthesis scaffolds and intrinsic ultrastructure were evaluated by histological staining and scanning electron microscopy (SEM), respectively. The distributions of collagen and PGs content in the decellularized book-shaped enthesis scaffolds from rabbit rotator cuff were also measured innovatively by SR-FTIR.

**Results:**

The decellularized book-shaped enthesis scaffolds from rabbit rotator cuff were successfully obtained. Histomorphology and SEM evaluated the effect of decellularization and the structure of extracellular matrix during decellularization. After mechanical testing, the failure load in the NET group showed significantly higher than that in the DEM group (*P* < 0.05). Meanwhile, the stiffness of the DEM group was significantly lower than the NET group. Furthermore, the distributions of collagen and PGs content in the decellularized book-shaped enthesis scaffolds were decreased obviously after decellularization by SR-FTIR quantitative analysis.

**Conclusion:**

SR-FTIR was applied innovatively to characterize the histological morphology of native enthesis tissues from rabbit rotator cuff. Moreover, this technology can be applied for quantitative mapping of the distribution of collagen and PGs content in the decellularized book-shaped enthesis scaffolds.

## Background

Bone-tendon interface (BTI), which is also named as enthesis, serves as an interface for force transmission from bone to tendon that consists of four transitional tissues: tendon, uncalcified fibrocartilage, calcified fibrocartilage, and bone [1, 2]. This transitional enthesis allows smooth transmission of forces derived from muscle contraction and minimizes formation of stress peaks [3, 4]. Enthesis injury, particularly those in the rotator cuff, are prevalent conditions that often lead to disability and persistent pain [5]. Regrettably, rapid and functional enthesis regeneration remains difficult because of its poor capacity of self-repair during healing [6–8]. Thus, conventional surgical treatment, only attaching the ruptured tendon and bony footprint together, cannot recapitulate the enthesis with graded and transitional structure, thus resulting in a high rate of re-rupture (20–94%) [1, 9].

With the development of tissue engineering technology, decellularized bioscaffolds have received great attention [10–12]. Previous studies indicated that organ-specific extracellular matrix scaffolds derived from site-specific homologous tissues may be better suited for constructive tissue remodeling than no site-specific tissue sources. As a result, decellularized extracellular matrix from enthesis may provide a natural three-dimensional scaffold with tissue-specific orientations of extracellular matrix molecules for enthesis regeneration. However, previously developed protocols for single tissue decellularization cannot be combined to prepare the decellularized enthesis scaffolds, as different tissues exhibit large differences in their components, microstructure characteristics and durability. Besides, the fibrocartilage region of enthesis is dense with low porosity, allowing for limited acellular solution infiltration, it is technically hard to remove its cellular components and antigens while mostly conserving native ECM. Thus, in this study, bone was treated together with fibrocartilage and tendons with different solutions in the same composite.

Histologically, BTI exhibits a gradual increase in collagen fiber organization moving from the bone to tendon [13]. The abundant type I collagen molecules are supplemented with type II, IX and XI collagen, and increased amount of proteoglycans is characteristic [14]. To date, Raman spectroscopy and Fourier transform infrared spectroscopic imaging (FTIR-I) have been used to analyze complex tissue transitions such as the ligament-to-bone [15] and tendon-to-bone [16, 17] interfaces. Recently, synchrotron radiation-based fourier transform infrared microspectroscopy (SR-FTIR) has been proved to be a useful tool for the analysis of biochemical quantification of collagen and proteoglycan content in biological samples [18]. In our previous studies, the SR-FTIR technology for quantitative mapping of the content and distribution of extracellular matrix in book-shaped decellularized fibrocartilage scaffold has been reported [19]. Zhou et al. found that SR-FTIR could be applied for quantitative evaluation of collagen and GAG in the cellularized or decellularized bioscaffolds [20]. The surgical outcome in small-animal experiments, such as mice, may not be replicable in large animals owing to differences in joint anatomy (structure) and function (mechanics) [3]. Furthermore, the healing ability of small animals is often faster than that of large animals or humans. Hence, the rabbit models of BTI healing that mimics the mechanical loading and healing ability of humans to evaluate a variety of new strategies for the treatment of rotator cuff tears is desirable [21]. Additionally, Mathewson et al. [22] compared rotator cuff muscle architecture of several animal models with that of humans and demonstrated that rabbit muscular parameters were more similar to humans than that in other large animals. However, to date, the application of SR-FTIR for quantitative evaluation of collagen and GAG in decellularized book-shaped enthesis scaffolds from rabbit rotator cuff has not been reported.

In this study, SR-FTIR was applied for quantitative mapping of the content and distribution of extracellular matrix in the decellularized book-shaped enthesis scaffolds from rabbit rotator cuff and verifying the decellularized method conducted by Su M et al’ protocol which could shorten decellularized time, well preserve the native structure, extracellular matrix components and mechanical properties of enthesis tissue [23].

## Methods

### Preparation of book-shaped bone-fibrocartilage-tendon samples

**Fig. 1:**
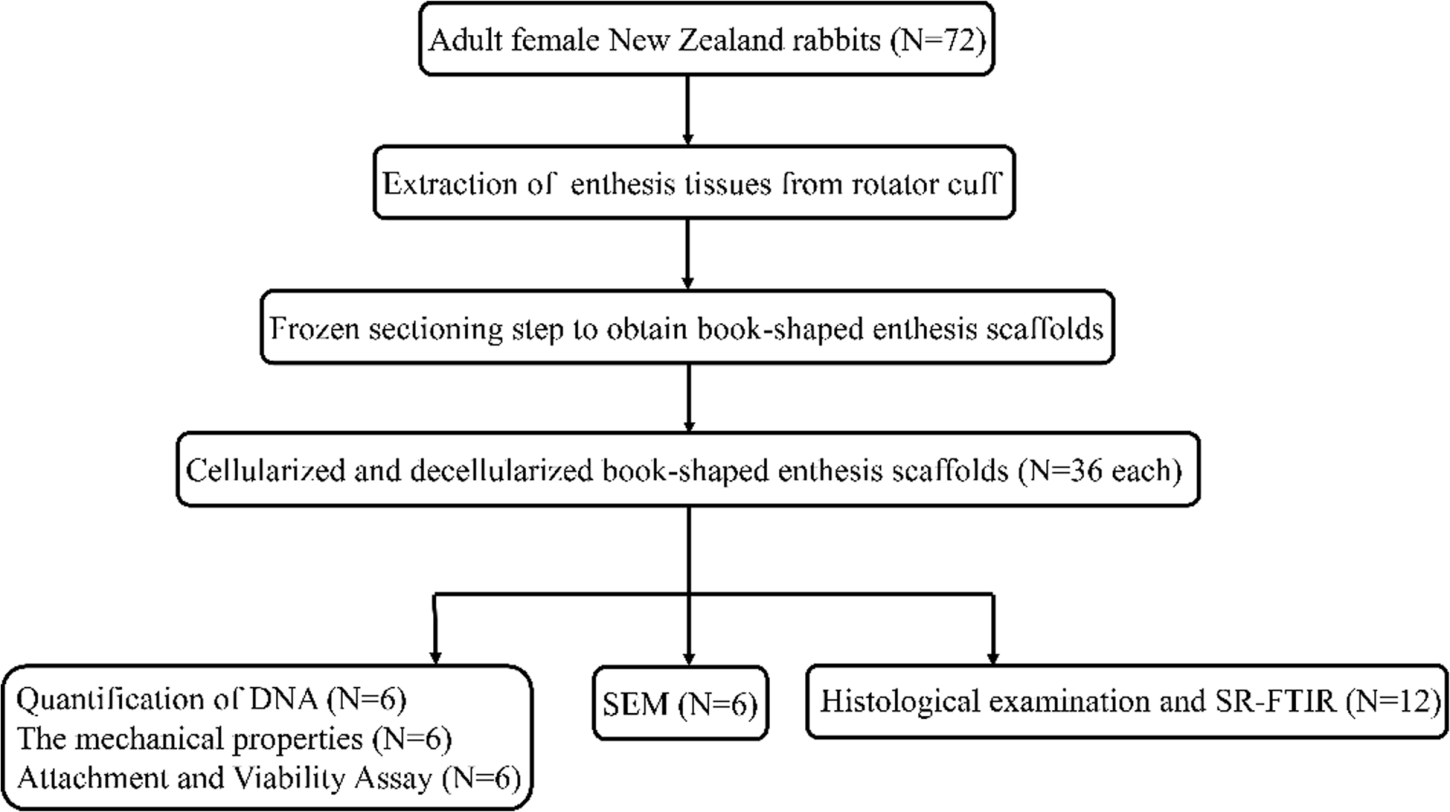
The technology roadmap of the experiment design

The whole experiment design is overviewed as in Fig. 1. The Ethics Committee of Xiangya Hospital, Central South University approved the research proposal (IRB: 2019030517). A total of 72 female New Zealand rabbits weighing 3.1 ± 0.3 kg were used in this study. After they were euthanized (intraperitoneal injection of sodium pentobarbital, 100 mg/kg), we obtained the native enthesis tissues (NET) specimens from rotator cuff, which were then sectioned into cuboid (about 30 mm × 1.2 mm × 10 mm) for decalcified and vertically sliced at the boundary between fibrocartilage and tendon. The specimens were sectioned into book shape from the tendinous end to bony end along motility direction with layer thicknesses of 250 μm.

### Preparation of decellularized book-shaped enthesis scaffolds

The decellularized book-shaped enthesis scaffolds were prepared as previously described by Su M et al’ protocol. Briefly, the samples were first immersed in liquid nitrogen for 2 min and then thawed in sterile PBS at 37 °C for 10 min, which was repeated five times. Then the samples were soaked in 1% Triton X-100 (Sigma-Aldrich, St. Louis, MO, USA) with agitation (100 rpm) for 1 day, followed by washing with 1% sodium dodecyl sulfate (SDS; Sigma-Aldrich) with agitation (100 rpm) for 1 day and ultrapure water for 1 day. Next, the tendon part of the sample was placed in a custom-designed container. The bone and fibrocartilage were exposed to 2% Triton X-100 for 4 days with agitation (100 rpm) and then washed in ultrapure water for 1 day, followed by washing with 3% SDS for 4 days with agitation (100 rpm) and ultrapure water for 1 day. Finally, the whole samples were treated with 100 U mL − 1 DNase I (Sigma-Aldrich) for 12 h. Finally, the book-shaped enthesis scaffolds were washed with ultrapure water for 1 day and embedded in paraffin for histological, and SR-FTIR examinations.

### Histology and scanning electron microscopy (SEM) analysis

Hematoxylin and eosin (H&E), toluidine blue fast green and 4′,6-diamidino-2-Phenylindole (DAPI) for histological observation was used to evaluate decellularized efficacy (*N* = 6). The decellularized book-shaped enthesis scaffolds from rabbit rotator cuff were embedded in paraffin and sectioned into 7 μm slices, then stained with HE, toluidine blue fast green and DAPI for observing the retention of nuclear materials. SEM image for the microstructure of the decellularized book-shaped enthesis scaffolds surface was observed and the decellularized components were detected (*N* = 6).

### SR-FTIR analysis

In this present study, the synchrotron radiation-Fourier transform infrared spectroscopy (SR-FTIR) we innovatively applied to evaluate the preservation of collagen and PGs in the NET and DEM (N = 6). The result of decellularization on the extracellular matrix components was evaluated using synchrotron radiation-Fourier transform infrared spectroscopy (SR-FTIR) at the BL01B beamline of National Facility for Protein Science Shanghai and Shanghai Synchrotron Radiation Facility, where synchrotron radiation from a bending magnet was collected, collimated and transported to a commercial FTIR interferometer bench. The peak area of amide I (1720–1590 cm-1) and carbohydrate (1140–985 cm-1) in the infrared spectrum were respectively calculated to characterize the distribution and content of collagen and PGs of the NET or DEM. The specific procedures are as previously described according to the published literature [13].

### DNA content analysis and mechanical tests

The quantifications of DNA in the decellularized book-shaped enthesis scaffolds were performed using DNeasy Blood & Tissue protocol according to the manufacturer’s instructions [12]. Specifically, the decellularized book-shaped enthesis scaffolds (*N* = 6) were weighed and minced after freeze-dried for 24 h using a lyophilizer (SIM International Group, USA). Then the decellularized bone-tendon scaffold (10 mg) was digested with proteinase K at 56 °C for 3 h. Finally, the DNA content in the decellularized book-shaped enthesis scaffolds was quantified by DNeasy Blood&Tissue Kit (Qiagen, USA) together with PicoGreen DNA assay kit (Invitrogen, USA).

The mechanical properties of the NET and DEM were comparatively evaluated with mechanical testing system (MTS insight, MTS Systems Corp, USA) for failure testing. Each specimen was preloaded to 1 N and then loaded to failure at a rate of 20 mm/min. Failure load and stiffness were calculated from the load-displacement curve obtained from the testing.

### Attachment and viability assay

After washing for three times with PBS (3 × 30 min), the decellularized book-shaped enthesis scaffolds were sterilized and immersed in a complete medium overnight, then 10^4^ BMSCs were respectively seeded onto the decellularized book-shaped enthesis scaffolds. To evaluate the cytotoxicity of the decellularized book-shaped enthesis scaffolds on BMSCs, cell viability was evaluated with a Live/Dead Assay kit (Invitrogen) at day 3 after seeding, Live/Dead assay showed that the green- and red-stained cells were captured by fluorescence with excitation wavelength of 488/594 nm to quantify cell viability (*N* = 6).

### Statistical analysis

The analyses were performed using the SPSS 25.0 software (SPSS, USA). All values were expressed as the mean ± standard deviation. Statistical significance of the experimental variables was then evaluated using Student’s t-test (*P* < 0.05 was considered statistically significant).

## Results

### Characterization of the decellularized book-shaped enthesis scaffolds by macroscopic observation and histomorphology

**Fig. 2:**
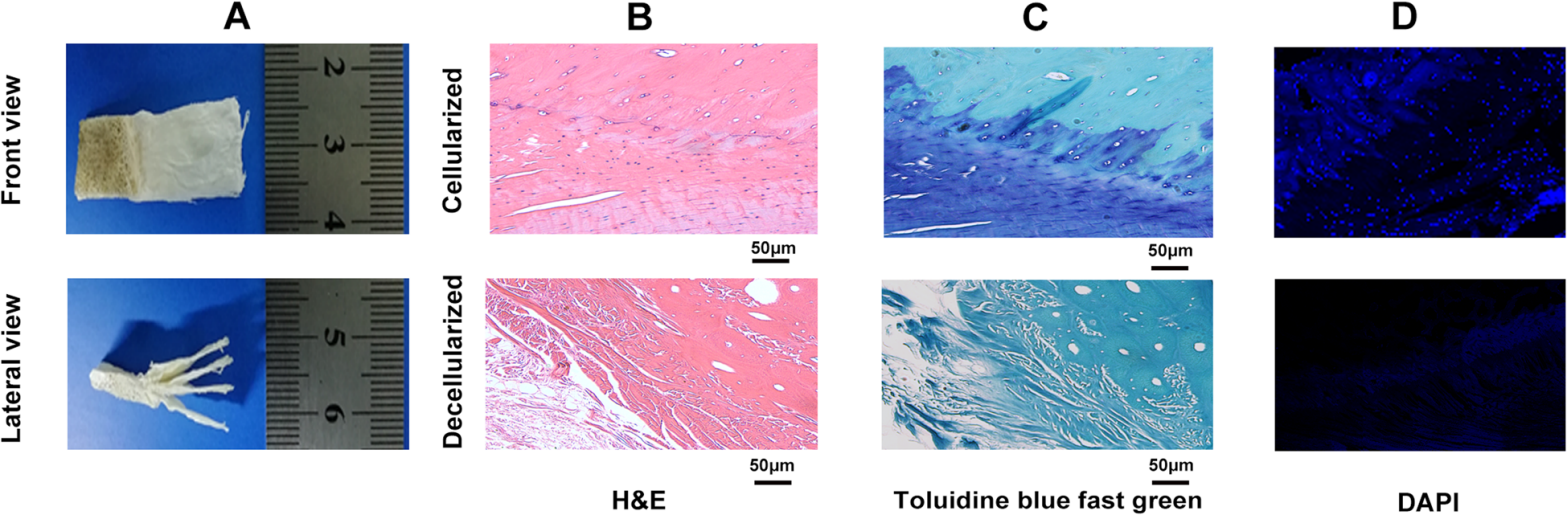
Characterization of the book-shaped enthesis scaffold. **a** The macroscopic observation of the decellularized book-shaped enthesis scaffold; **b** H&E staining, **c** Toluidine blue fast green staining, and **d** DAPI staining were used together to evaluate the decellularized effect in the decellularized book-shaped enthesis scaffold. Bar = 100 μm. UCF: uncalcified fibrocartilage, CF: calcified fibrocartilage

In this study, enthesis tissue was sectioned into “book” shape along motility direction with thickness of 250 um (Fig. 2a). H&E, Toluidine blue fast green, Dapi staining were used together to evaluate the decellularized effect. H&E and Toluidine blue fast green staining showed that the cellular components of the decellularized book-shaped enthesis scaffolds were absolutely removed, while the structure and morphology of the native enthesis extracellular matrix were well preserved (Fig. 2b, c). Besides, Dapi-positive cell nuclei were rarely shown in the decellularized book-shaped enthesis scaffolds (Fig. 2d).

### SEM analysis

**Fig. 3:**
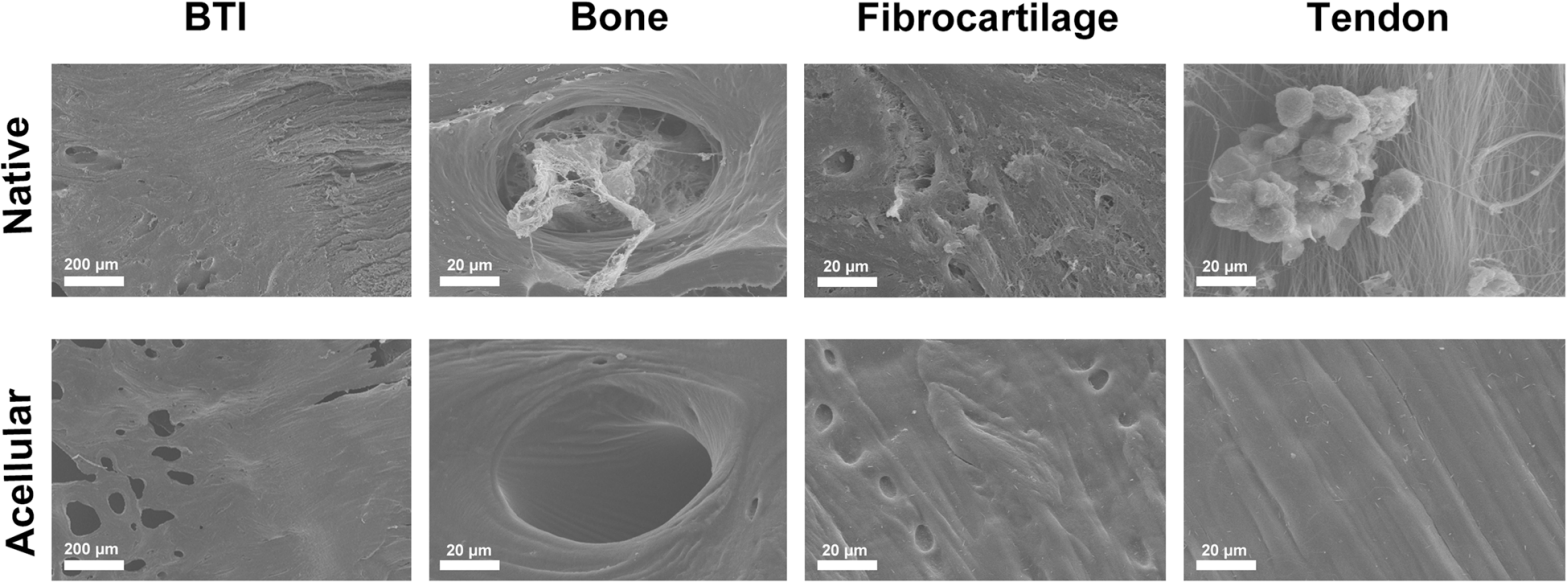
From the SEM image, the decellularized book-shaped enthesis scaffolds following Su M et al’ protocol preserved native collagen structure well, and no cells debris was visualized

The surface topology of book-shaped enthesis scaffolds before or after decellularization was detected by SEM (Fig. 3). In the native book-shaped enthesis scaffolds, cells adhered to various native tissues. After decellularization, we observed no cellular components on the surface of the scaffold.

### Distribution and content of collagen and proteoglycan by SR-FTIR

**Fig. 4:**
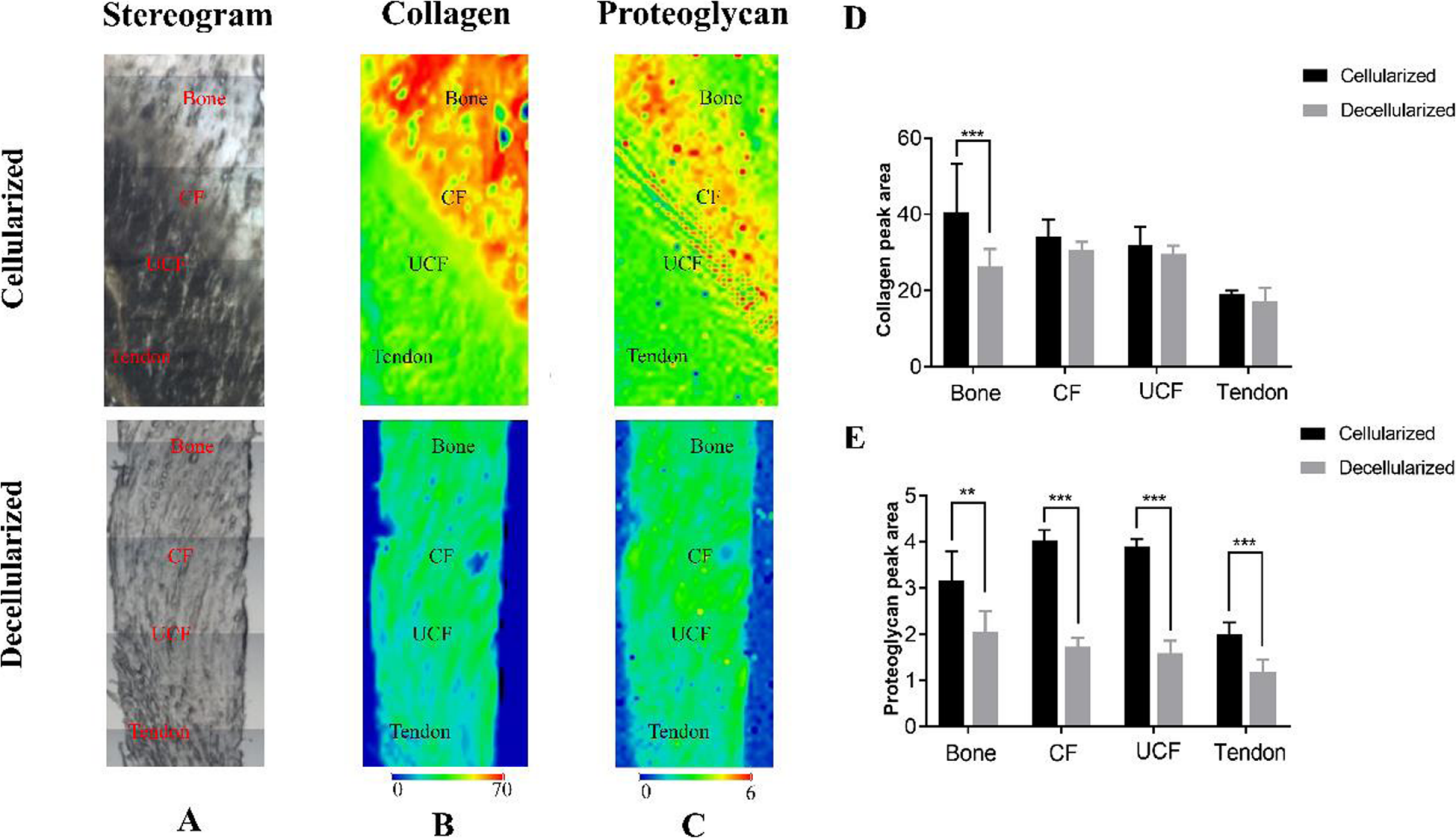
SR-FTIR mappings (Scale bar: 100 μm), including collagen and proteoglycan distribution, of the NET or DEM. **a** Light microscopy images. **b** Distribution of collagen. **c** Distribution of proteoglycan. **d** Content of collagen within the cellularized or decellularized book-shaped enthesis scaffolds. **e** Collagen and GAGs contents in the Bone, CF, UCF or Tendon region within the cellularized or decellularized book-shaped enthesis scaffolds. *, *P* < 0.05; **, *P* < 0.01; ***, *P* < 0.001

Collagen and proteoglycan contents were evaluated by integrating the peak area under the Amide I band (1720–1590 cm-1) and C-O-C and C-OH vibrations (1140–985 cm-1), respectively. In this study, we innovatively applied SR-FTIR to comparatively characterize the distribution and content of collagen and proteoglycan between NET and DEM. For each sample, regions of interest (~ 750 × ~1750um/region) containing tendon, fibrocartilage, and bone were scanned, and ~ 8000 points of spectral data were acquired per region, constituting a total of ~ 24,000 spectra collected per sample. As presented in Fig. 4 (*N* = 6), the collagen content in the bone regions lost about 40.85%, while the collagen contents in the CF (calcified fibrocartilage), UCF (uncalcified fibrocartilage) and tendon regions of DEM were similar without significant difference between NET and DEM samples (Fig. 4d). Meanwhile, SR-FTIR analysis indicated that the PGs distribution at DEM was partly reserved after decellularization, its content decreased about 44.55, 56.76, 58.13 and 46.33% in the bone, CF, UCF and tendon regions of NET, respectively (Fig. 4e).

### DNA residence and mechanical test

**Fig. 5:**
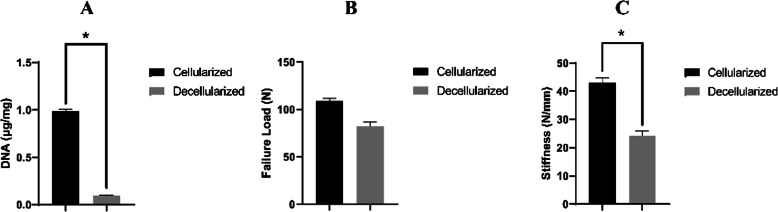
Quantification of DNA, failure load, and stiffness in the decellularized book-shaped enthesis scaffolds. **a** Content of DNA within the NET or DEM. **b** The mechanical properties (failure load). **c** The mechanical properties (stiffness). Dissimilar letters indicating a significant difference (*P* < 0.05)

After decellularization, the content of DNA was greatly reduced (0.089 ± 0.018 μg/mg) in the DEM, which was significantly lower than that in the NET (0.976 ± 0.026 μg/mg) (*P* < 0.05) (Fig. 5a). After mechanical testing, the failure load in the NET group showed significantly higher than that in the DEM group (*P* < 0.05) (Fig. 5b). Meanwhile, the stiffness of the DEM group was significantly lower than the NET group (Fig. 5c).

### Biological characteristics of DEM on BMSCs viability

**Fig. 6:**
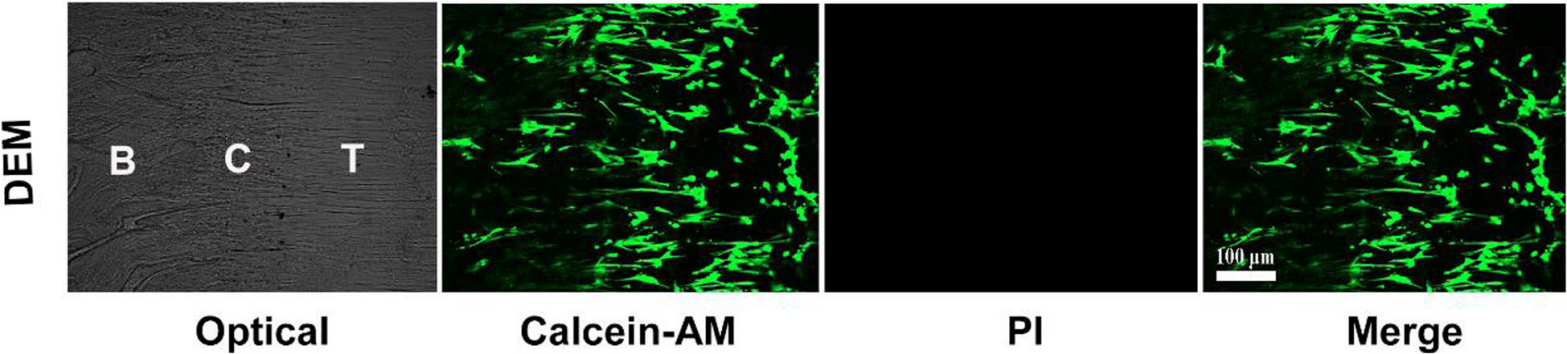
Live/dead cell analysis was used to observe the cell viability of the decellularized book-shaped enthesis scaffolds. Live/dead double staining after the hBMSCs seeded on the DEM for 3 days (green fluorescence represented live cells, while red for dead). (*N* = 6). Scale bar: 100 μm

To evaluate the effects of DEM on BMSCs viability, Live/Dead assay was used to examine cell proliferation and viability of BMSCs cultured on the DEM. At day 3 after seeding, Live/Dead assay showed that most BMSCs were stained fluorescent green (living cells), with very few red (dead cells) (Fig. 6).

## Discussion

The rotator cuff repair, which often leads to disability and persistent pain, is very common in the shoulder and the bone-tendon interface healing is crucial to regenerate native fibrocartilaginous structure. However, this unique tissue structure healing remains difficult because of the limited ability of tendons to self-repair [6]. To improve the healing of bone-tendon interface, tissue engineering has been widely examined in this field, utilizing a combination of scaffolds, bioactive molecules and seeded cells to repair damaged tissues.

Decellularization technology has been widely used to obtain decellularized scaffolds, such as decellularized bone, fibrocartilage, or tendon tissue. There are two crucial steps during decellularization: removing cell components and preserving the extracellular matrix components. Collagen and proteoglycan are two important biocomponents in the extracellular matrix and the main structures of bone, fibrocartilage, and tendon extracellular matrix are composed of different types of collagen [24]. In our previous study, Chen et al. applied book-shaped decellularized fibrocartilage scaffold for bone-tendon healing in patella patellar-tendon complexes and achieved better results [19]. Nevertheless, few decellularization methods, which not only well removed cell component and preserved the whole natural structure and mechanical properties, is available for the large-size enthesis scaffold. Recently, Su M et al. developed a decellularization protocol for porcine enthesis decellularization, which includes the processes of tissue-trimming, freeze-thaw cycles, 3%SDS, and nuclease digestion. However, whether the contents and distribution of collagen fiber and proteoglycan could be reserved still needs to be explored.

With the development of the third generation synchrotron light source Shanghai Synchrotron Radiation Facility (SSRF), the SR-FTIR (higher spatial resolution of 5 μm) has been applied to analysis at the diffraction limit while preserving a high spectral quality [13]. The flux at the entrance of the SR-FTIR spectrometer has been achieved to be about 1.5 × 10^13^ (photons/sec/0.1% b.w.) at 1 μm wavelength for a 230 mA current. These performances allow the SR-FTIR to analyse large samples with heterogeneous regions in a small area with a diffraction limited spatial resolution [25]. This study utilizes SR-FTIR as it is a high-throughput and sensitive imaging modality which is capable of quantitative mapping of the content and distribution of extracellular matrix, thereby making it well suited for examining multi-tissue regions. Unlike those of traditional histological staining techniques and biochemical assays, the SR-FTIR technique can be used to quantitatively map decellularized book-shaped enthesis scaffolds. Compared with the conventional FTIR, the SR-FTIR can detect the changes in microstructure in high resolution, and determine the various components.

Meanwhile, utilizing SR-FTIR technique, both collagen, proteoglycan, as well as collagen orientation, were detected and quantified across the different regions of the decellularized book-shaped enthesis scaffolds. The extent of decellularization on extracellular matrix of the bone-tendon scaffolds can be assessed by such structural chemical information. Evaluating these compositional changes is crucial to elucidating the role of the bioscaffolds in rabbit rotator cuff [15]. In this study, SR-FTIR was applied for quantitative mapping of the content and distribution of extracellular matrix in the decellularized book-shaped enthesis scaffolds from rabbit rotator cuff for the first time. As shown in our results, 40.85% collagen and 44.55% proteoglycan is lost after decellularization. Our findings provide critical information for the regeneration of bone-tendon interface and new insights into matrix composition and organization across the decellularized bone- tendon scaffold. To prove this interpretation, we should increase the number of samples and study other types of BTI in follow-up experiments.

This present study focused on quantitative mapping of changes in matrix components across the decellularized large-size enthesis tissue as scaffolds using the SR-FTIR technology. Assessing the matrix components of decellularized meniscus and cartilage extracellular matrix by the SR-FTIR technology are the next steps in the following studies. The future application of SR-FTIR would be extended to observe mineral distributions and potentially facilitate the understanding of the transition of complex loads from bone to ligament. However, there were still a few limitations that remained in the current study. Firstly, there are some differences in the biomechanics and size of the repair region between rabbits and humans. Pre-clinical animals such as canine or goat should be performed in the next step before clinical usage. Furthermore, comparison studies between FTIR and SR-FTIR in assessing matrix components of decellularized book-shaped enthesis scaffolds still needed further investigation.

## Conclusion

In summary, the goal of this study is to utilize SR-FTIR to analyze the distributions of collagen and PGs content in the decellularized book-shaped enthesis scaffolds from rabbit rotator cuff. After following Su M et al’ decellularization protocol, cell components were effectively removed and the microstructure of the scaffold was well preserved, however, changes in extracellular matrix components, such as collagen and proteoglycan, were observed.

## Data Availability

The datasets used and/or analysed during the current study are available from the corresponding author on reasonable request.

## Abbreviations

BMSC: Bone marrow stromal cell
OCT: Optimum cutting temperature
SEM: Scanning electron microscopy
SR-FTIR: Synchrotron radiation-based Fourier transform infrared microspectroscopy
SSRF: Shanghai Synchrotron Radiation Facility
NET: Native enthesis tissues
DEM: Decellularized enthesis matrix
BTI: Bone-tendon interface
